# Assessment of the Quality of Newly Formed Bone around Titanium Alloy Implants by Using X-Ray Photoelectron Spectroscopy

**DOI:** 10.1155/2012/615018

**Published:** 2012-06-18

**Authors:** Hiroshi Nakada, Toshiro Sakae, Yasuhiro Tanimoto, Mari Teranishi, Takao Kato, Takehiro Watanabe, Hiroyuki Saeki, Yasuhiko Kawai, Racquel Z. LeGeros

**Affiliations:** ^1^Department of Removable Prosthodontics, Nihon University School of Dentistry at Matsudo, 2-870-1 Sakaecho-Nishi Matsudo City, Chiba 271-8587, Japan; ^2^Research Institute of Oral Science, Nihon University School of Dentistry at Matsudo, 2-870-1 Sakaecho-Nishi Matsudo City, Chiba 271-8587, Japan; ^3^Department of Histology, Nihon University School of Dentistry at Matsudo, 2-870-1 Sakaecho-Nishi Matsudo City, Chiba 271-8587, Japan; ^4^Department of Dental Biomaterials, Nihon University School of Dentistry at Matsudo, 2-870-1 Sakaecho-Nishi Matsudo City, Chiba 271-8587, Japan; ^5^Department of Oral Implantology, Nihon University School of Dentistry at Matsudo, 2-870-1 Sakaecho-Nishi Matsudo City, Chiba 271-8587, Japan; ^6^Department of Biomaterials and Biomimetics, New York University College of Dentistry, 345 East 24th Street, New York, NY 10010, USA

## Abstract

The aim of this study was to evaluate differences in bones quality between newly formed bone and cortical bone formed around titanium alloy implants by using X-ray photoelectron spectroscopy. As a result of narrow scan measurement at 4 weeks, the newly formed bone of C1s, P2p, O1s, and Ca2p were observed at a different peak range and strength compared with a cortical bone. At 8 weeks, the peak range and strength of newly formed bone were similar to those of cortical bone at C1s, P2p, and Ca2p, but not O1s. The results from this analysis indicate that the peaks and quantities of each element of newly formed bone were similar to those of cortical bone at 8 weeks, suggestive of a strong physicochemical resemblance.

## 1. Introduction

Dental implantation is a treatment method in which fixtures are implanted in the jawbone, followed by prosthetic implantation after a resting period of approximately 3–6 months, at which time new bone is formed around the fixtures [1]. This process sets the cortical bone as the primary anchorage unit. Bone modeling and remodeling processes are important (1) to induce long-term stability of the implants; (2) to develop osseointegration between implant materials and the bone; (3) to allow the maturation of new bone around the implants. It has been reported that the maximum occlusal force in adults with a natural dentition is 430 N [2], and similar loads are likely to be applied to implants as well as normal prostheses. Therefore, to achieve long-term retention and stability of implants under such conditions, the quality of newly formed bone around implants is important.

Many studies have reported that newly formed bone around implants is spongy bone [3]. However, although the morphology of newly formed bone is reportedly like spongy bone, it is difficult to discriminate whether the bone quality is mature like cortical bone, or immature like spongy, osteoid, or cartilaginous bone; therefore, evaluation of the bone quality is required. 

The quality of bone forming around implants has been investigated by various groups. Nakano et al. [4] evaluated bone density and alignment of biological apatite (BAp), and Boskey and Pleshko [5] used Fourier-transform infrared (FTIR) imaging to assess bone and cartilage quality and composition. In addition, our group has reported on the use of polarized microscopy [6] and scanning electron microscopy (SEM) [7] to evaluate new bone and cortical bone quality, as well as microscopic Raman spectroscopy to analyze phosphate peaks of bone apatite [6], and microcomputed tomography (micro-CT) to assess trabecular microarchitecture and bone mineral density (BMD) [8–10]. However, basic research on new bone formation has been sparse, and molecular and elemental characterization of BAp, the basic building block of bone, has generally been overlooked. Moreover, responses to early dynamic loading in implants, with analysis of changes in new bone quality associated with mineralization, remain an important issue for further research.

In this study, differences in the bone quality between newly formed bone and cortical bone formed around titanium (Ti) alloy implants were investigated using X-ray photoelectron spectroscopy (XPS).

## 2. Materials and Methods

### 2.1. Experimental Animals 

Six 18-week-old New Zealand White Rabbits (Sankyo Labo Service Co., Tokyo, Japan) were used in experiments. The rabbits were housed in individual metal cages at a room temperature of 23 ± 1°C and humidity of 50 ± 1%, with ad libitum access to food and water. The experimental protocol was approved by an animal experimentation ethics committee (approval number ECA 07-0016). All experiments were conducted according to the Guidelines for the Treatment of Animals, Nihon University, Chiba, Japan.

### 2.2. Materials

**Figure 1:**
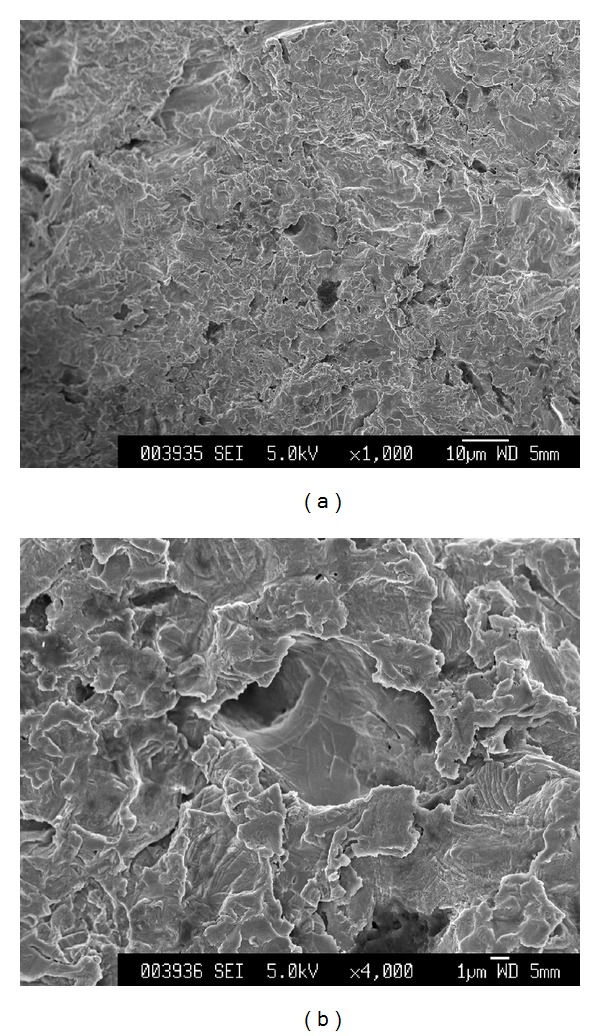
Scanning electron microscope photographs of implant surface at (a) low magnification and (b) high magnification.

Implants (diameter, 3.0 mm; length, 7.0 mm) were fabricated using Ti-15%Zr-4%Nb-4%Ta (Ti-15-4-4) alloy [11–15] (Table 1 displays its chemical composition). Surface treatment of implants consisted of sulfuric acid-etching and grit-blasting with apatitic abrasive (particle size 250 *μ*m, HiMed Co., NY, USA) (Figure 1).

### 2.3. Implantation 

Rabbits underwent general anesthesia with 2.0 mg/kg of intravenous Ketalar (Daiichi Sankyo, Tokyo, Japan). Implant cavities were surgically created in the tibia 10 mm distal to the knee joint, one each bilaterally, by using a 1.0 mm and 3.0 mm diameter round bar, while irrigating the area with sterile saline. Implants were inserted into the right tibia, with each rabbit receiving one implant (8 implants were used in total). After surgery, the areas were disinfected with tincture of iodine for 3 days. Rabbits were sacrificed by anesthesia overdose at 4 or 8 weeks after-implantation, and the tibias were resected.

### 2.4. XPS Analysis 

**Figure 2:**
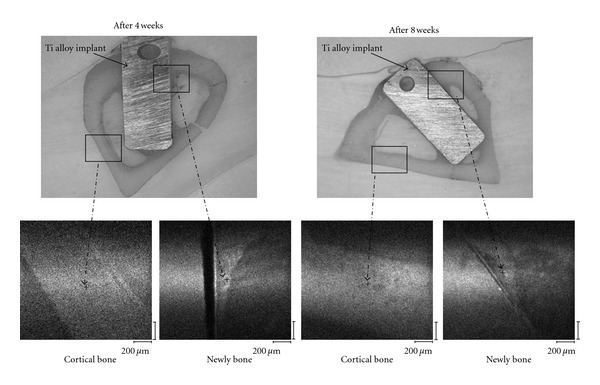
Measurement areas for XPS analysis (upper: thin section specimens; lower: enlarged images; +: measurement areas). Newly bone: newly formed bone.

For XPS analysis, newly formed bone in close proximity to implants and cortical bone (control) which not in close proximity to implants were analyzed using thin-cut nondecalcified histological specimens. Since both bone resorption by osteoclasts and bone formation by osteoblasts progress concurrently, differences in measurement of bone tissue results are apt to occur; therefore, it is difficult to specify the measurement area. In the present study, more than 3 areas of newly formed bone close to implants and those of cortical bone were measured, and sites within which appropriate average values were obtained were determined as the measurement areas (Figure 2). An XPS analysis was performed under the following conditions: X-ray source: monochromatic AlK*α* (1,486.6 eV), detection region: 20 *μ*m*θ*, and detection depth: approximately 4-5 nm (take-off angle: 45°).

Qualitative analysis was performed using wide scan measurement, and the chemical bonding conditions of detected elements were analyzed using narrow scan measurement. Hydroxyapatite powders were used as the standard specimens, followed by performance of measurements with a correction for relative sensitivity factors (RSFs).

## 3. Results

**Figure 3:**
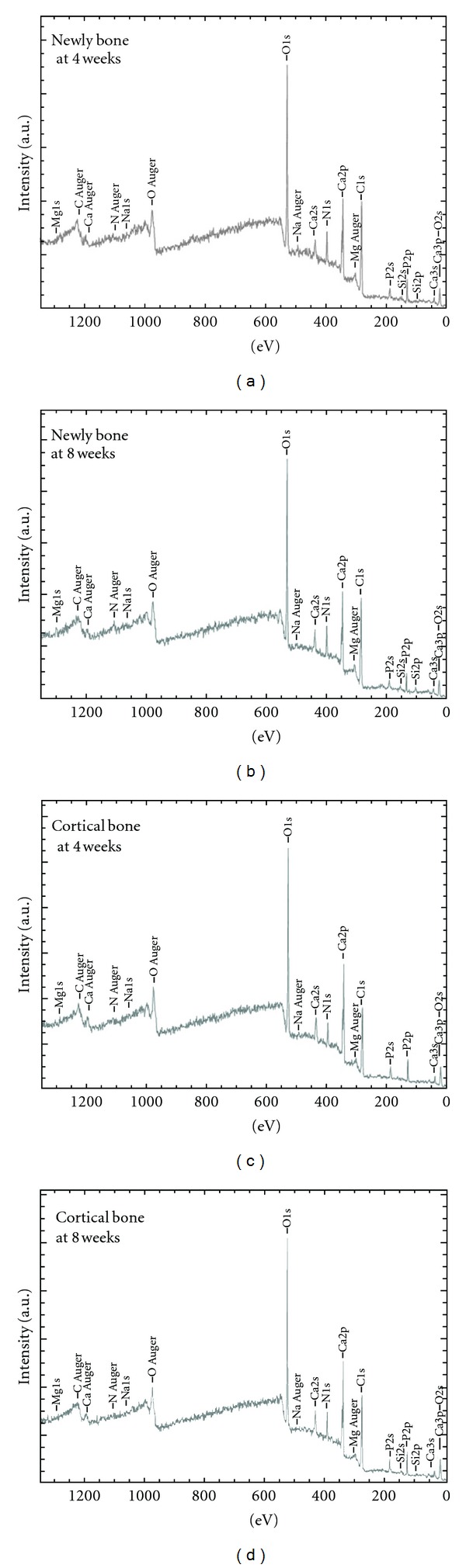
Wide scan peaks of each element at 4 and 8 weeks. Newly bone: newly formed bone.

**Figure 4:**
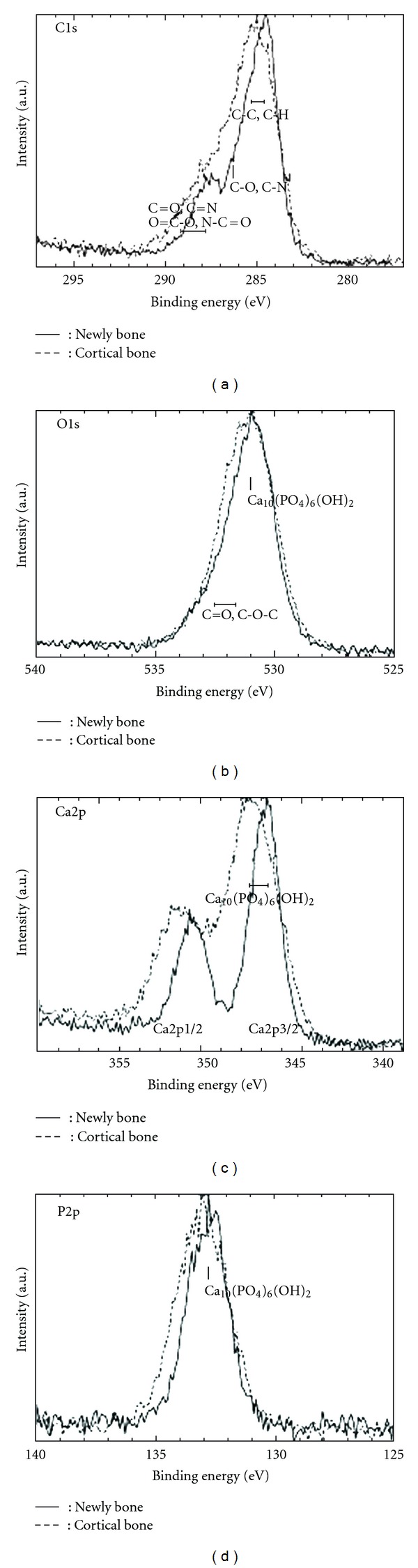
Narrow scan peaks of each element at 4 weeks. Newly bone: newly formed bone.

**Figure 5:**
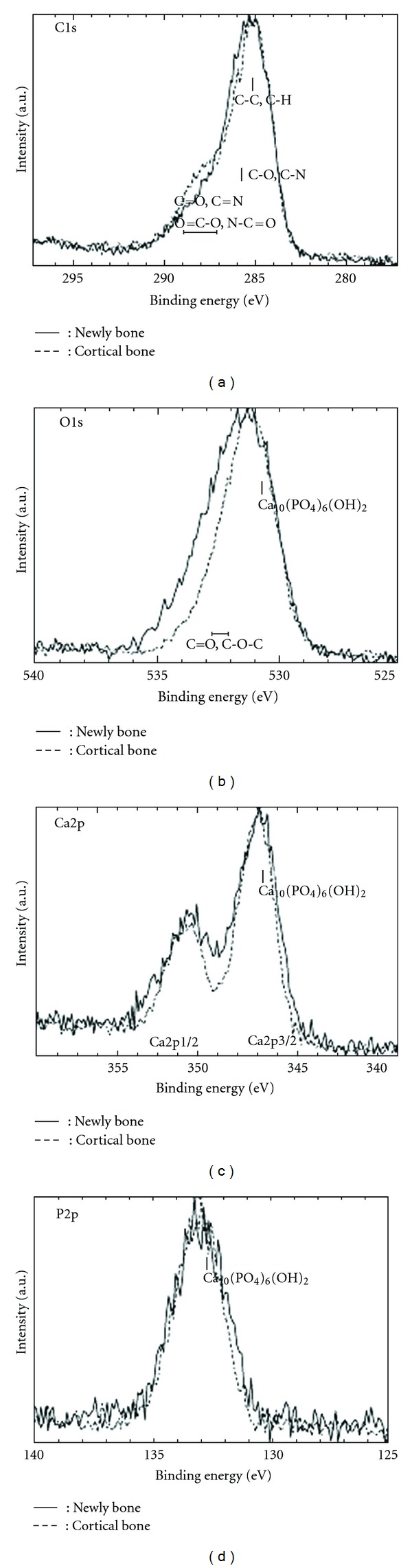
Narrow scan peaks of each element at 8 weeks. Newly bone: newly formed bone.

Qualitative analysis of the peak strengths on newly formed bone around implants and cortical bone at 4 and 8 weeks is shown in Figure 3. Although peaks of Ca, O, P, C, Mg, N, and Na were detected, a marked unevenness in these peaks was observed at 4 weeks, in contrast to 8 weeks. Based on the results of wide scan measurement, narrow scan measurement of elements related to Ca_10_(PO_4_)_6_OH_2_ was performed. The resulting overlap of C1s, O1s, Ca2p, and P2p of newly formed and cortical bone at 4 weeks is shown in Figures 3 and 4, respectively; the results at 8 weeks are shown in Figure 5.

The results of narrow scan measurement at 4 weeks (Figure 4) indicate that, although the peak strengths of newly formed and cortical bone were almost equal at O1s, the half-width became smaller in newly formed bone. At P2p and C1s, although a shifting of the peak in newly formed bone was observed, the half-width became smaller in newly formed bone; the peak strength of newly formed bone at C1s were lower than that of cortical bone. At Ca2p (Ca2p 3/2), although the peak strengths of newly formed bone was lower than that of cortical bone, the half-width became smaller in newly formed bone, and a shifting of the peak was observed. The chemical bonding condition observed for each element in newly formed bone differed from that of cortical bone.

As a result of narrow scan measurement at 8 weeks (Figure 5), the half-width and strength of newly formed bone were almost equal to those of cortical bone at C1s, P2p, and Ca2p. At O1s, the half-width became small in newly formed bone, and a shifting of the peak was observed. The chemical bonding condition observed for each element of newly formed bone was similar to that of cortical bone.

Table 2 shows the results of the quantitative analysis of each element and the Ca/P ratio. The results for newly formed bone were Ca: 15.07 ± 2.83 weight percent and P: 7.83 ± 1.56 weight percent at 4 weeks; Ca: 17.33 ± 2.393 weight percent and P: 8.90 ± 0.80 weight percent at 8 weeks. These values gradually became similar to those of cortical bone at 4 weeks (Ca: 17.33 ± 2.39 weight percent and P: 8.90 ± 0.80 weight percent) and at 8 weeks (Ca: 21.33 ± 1.88 weight percent and P: 11.03 ± 0.70 weight percent).

## 4. Discussion

The use of XPS analysis in the present experiment has been employed in industry since the 1960s and is now being clinically applied to the qualitative analysis of several nanometer-sized areas on material surfaces. Elemental analysis showed complex spectra based on chemical bond arrangement and elemental compositional changes. Changes in spectra reveal alterations in chemical bonds based on changes in interatomic transition and valence-band state density.

In the present study, newly formed bone at 4 weeks showed differences in the Ca/P ratio (Table 2), and in peak position, peak height, and half-width on the narrow scan measurement of each element (Figures 4 and 5). Many mineral elements were present in newly formed bone at 4 weeks, suggesting that the arrangement of chemical bonding and element composition in newly formed bone differed from those in cortical bone. Furthermore, the peak position, peak height, and half-width on narrow scan measurement at 8 weeks in newly formed and cortical bone showed similar traces, and the quantitative analysis of new and cortical bone at 8 weeks (Table 2) showed similar results. These results suggest that newly formed bone at 8 weeks showed a similar bone quality to that of cortical bone, due to progressive bone maturation and bone metabolism of minerals.

Each element showed a complex spectrum with changes in the arrangement of chemical bonding regarding the peak and element composition. The spectrum is related to changes in the chemical bonding of elements caused by interatomic transition and changes in the density or state of the valence band. Under the atomic arrangement of BAp crystals in immature new bone, various minor and trace elements, including CO_3_^2−^, Na^+^, and Mg^2+^, were substituted for Ca^2+^, PO_4_^3−^, and OH^−^. Such substitutions have been shown to affect the properties of BAp crystals [16]. For example, substitution of CO_3_^2−^ for PO_4_^3−^ in the apatite lattice can cause changes in chemical bonding that leads to strain and reduced crystallite size in BAp [16, 17]. Such changes in chemical bonds can result in changes in binding energy in the magnitude of several electron volts (eV). In chemical bonds, trace element peaks shift to higher energy as differences in the electronegativity of bond-forming elements and the electron valence of elements increase. Peak shifts are easily influenced by the surrounding molecular environment, and peak shift and half-width variations signify the presence of compounds with different molecular weights and atomic arrangement. The peak value in a chemical bond is clear with many reports [18–21]. The main combined states of each element and a corresponding peak are shown in Table 3.

In the present study, differences were observed in XPS spectra between new bone at 4 weeks postimplantation and cortical bone, indicating immature bone quality due to BAp imperfection. In the chemical bonds of each spectrum, changes in the order of 0.1 eV affected BAp crystallinity [22].

The XPS spectra of new bone at 8 weeks postimplantation resembled those of cortical bone, and quantitative analysis showed higher Ca and P in newly formed bone compared to 4 weeks. This indicated that the composition of newly formed bone was closer to that of cortical bone at 8 weeks. Osseointegration around implants occurs as mineralization progresses, involving the accumulation of mineral components or BAp nanocrystals [23]. However, the present study was unable to analyze in detail the chemical bonding present in BAp nanocrystals, a subject for future analysis. 

## Figures and Tables

**Table 1:** Chemical composition (mass %) of the Ti-15-4-4 alloy used in the present study.

Titanium alloy	(mass %)									
	Zr	Nb	Ta	Pd	Fe	O	N	H	C	Ti
Ti-15-4-4	15.24	3.90	3.92	0.22	0.022	0.162	0.048	0.011	0.002	Bal.^∗∗^

^∗∗^Bal.: balance.

**Table 2:** Results of the quantitative analysis of newly formed bone around implants at 4 and 8 weeks, and cortical bone. Newly bone: newly formed bone.

	(Weight %)		
	Ca	P	Ca/P
4 weeks newly bone	15.07 ± 2.83	7.83 ± 1.56	1.93 ± 0.10
4 weeks cortical bone	19.77 ± 1.65	10.27 ± 1.05	1.93 ± 0.07
8 weeks newly bone	17.33 ± 2.39	8.90 ± 0.80	1.94 ± 0.10
8 weeks cortical bone	21.33 ± 1.88	11.03 ± 0.70	1.93 ± 0.05

**Table 3:** Main chemical bonding of each element and peak position (eV).

Peak	Binding energy (eV)	Peak position
	Metal—O	530-531
O1s	Metal—PO*x*, −OH	531-532
	C=O, C–O–C	532-533

	C–C, C–H	284.8
C1s	C–O, C–N	286
	C=O	287-288
	O=C–O	289

	Ca	346
Ca2p 3/2	CaO	346-347
	Ca_10_ (PO_4_)_6_ (OH)_2_	346-347

	Metal—P	129
P2p	Metal—PO*x *	133.5
	P_2_O_5_	135
	Ca_10_ (PO_4_)_6_ (OH)_2_	133–133.5

	C–N, C=N, N–H	398.5–401
Nls	Metal–N	396–398
	NO	401–403

	Si	99.4
Si2p	SiO*x*, SiOC	101–103
	SiO_2_	103–103.6
